# Enhancing Seasonal Influenza Surveillance: Topic Analysis of Widely Used Medicinal Drugs Using Twitter Data

**DOI:** 10.2196/jmir.7393

**Published:** 2017-09-12

**Authors:** Ireneus Kagashe, Zhijun Yan, Imran Suheryani

**Affiliations:** ^1^ School of Management and Economics Beijing Institute of Technology Beijing China; ^2^ Sustainable Development Research Institute for Economy and Society of Beijing Beijing China; ^3^ School of Life Science Department of Biomedical Engineering Beijing Institute of Technology Beijing China

**Keywords:** machine learning, Twitter messaging, social media, disease outbreaks, influenza, public health surveillance, natural language processing, influenza vaccines

## Abstract

**Background:**

Uptake of medicinal drugs (preventive or treatment) is among the approaches used to control disease outbreaks, and therefore, it is of vital importance to be aware of the counts or frequencies of most commonly used drugs and trending topics about these drugs from consumers for successful implementation of control measures. Traditional survey methods would have accomplished this study, but they are too costly in terms of resources needed, and they are subject to social desirability bias for topics discovery. Hence, there is a need to use alternative efficient means such as Twitter data and machine learning (ML) techniques.

**Objective:**

Using Twitter data, the aim of the study was to (1) provide a methodological extension for efficiently extracting widely consumed drugs during seasonal influenza and (2) extract topics from the tweets of these drugs and to infer how the insights provided by these topics can enhance seasonal influenza surveillance.

**Methods:**

From tweets collected during the 2012-13 flu season, we first identified tweets with mentions of drugs and then constructed an ML classifier using dependency words as features. The classifier was used to extract tweets that evidenced consumption of drugs, out of which we identified the mostly consumed drugs. Finally, we extracted trending topics from each of these widely used drugs’ tweets using latent Dirichlet allocation (LDA).

**Results:**

Our proposed classifier obtained an F_1_ score of 0.82, which significantly outperformed the two benchmark classifiers (ie, *P*<.001 with the lexicon-based and *P*=.048 with the 1-gram term frequency [TF]). The classifier extracted 40,428 tweets that evidenced consumption of drugs out of 50,828 tweets with mentions of drugs. The most widely consumed drugs were influenza virus vaccines that had around 76.95% (31,111/40,428) share of the total; other notable drugs were Theraflu, DayQuil, NyQuil, vitamins, acetaminophen, and oseltamivir. The topics of each of these drugs exhibited common themes or experiences from people who have consumed these drugs. Among these were the enabling and deterrent factors to influenza drugs uptake, which are keys to mitigating the severity of seasonal influenza outbreaks.

**Conclusions:**

The study results showed the feasibility of using tweets of widely consumed drugs to enhance seasonal influenza surveillance in lieu of the traditional or conventional surveillance approaches. Public health officials and other stakeholders can benefit from the findings of this study, especially in enhancing strategies for mitigating the severity of seasonal influenza outbreaks. The proposed methods can be extended to the outbreaks of other diseases.

## Introduction

### Background

Public health surveillance involves the systematic collection, management, analysis, and interpretation of health-related data, followed by the dissemination of these data to public health programs to enhance public health actions [1,2]. This process comes in handy during periods of severe health concerns such as disease outbreaks that need preventive and control interventions.

Among these disease outbreaks is influenza-like illness (ILI) or flu, which is a respiratory illness caused by viruses that can cause severe illness and even death for some people [3]. Common influenza types include seasonal, avian, swine, variant, and pandemic. Due to its recurring nature, we focused on seasonal influenza in this study.

To control disease outbreaks of seasonal influenza, one widely adopted measure is the proper use of medicinal drugs (ie, drugs which treat, prevent, or alleviate symptoms of diseases) [4]. When outbreaks occur, it is vitally important to know and analyze feedback data related to drugs that are widely consumed by people so that control measures to fight these outbreaks can be enhanced and implemented in the future.

Our work is built on the knowledge that seasonal influenza outbreaks affect a large number of people and are spread over large geographical areas. These facts pose challenges in gathering and analyzing useful feedback data related to the consumption of medicinal drugs by using traditional or conventional surveillance methods, which are limited by a small sample size, cost, and timeliness in reporting [5-7].

Therefore, this research study intended to extract relevant topics from tweets mentioning widely consumed drugs during seasonal influenza outbreaks and to use those topics to enhance seasonal influenza surveillance.

With the fast development of Web 2.0 technology, we considered using social media because of its ability to collect vast amounts of health-related data. Twitter, which was established in 2006, is among the most famous social media platforms and is currently the leading microblogging service for people to send and receive messages (tweets) of up to 140 characters. Twitter has a volume of 313 million monthly active users, 1 billion unique monthly visits, and 500 million tweets per day [8]. The communication forms in Twitter can be chats, conversations, news reporting, and information sharing [9]. People discuss their health conditions and statuses on Twitter [10], which makes it a new potential data source for health-related studies examining the prevalence of health issues, drug consumption, and health topics or categories [11-19].

To follow the use of influenza drugs in this study, we could have followed sales of over-the-counter and prescription drugs [5,20], or we could have used search engine queries and Web data [6,7,21,22]. However, prescription drug sales would not allow us to obtain topics or feedback from consumers, and access to these data is limited, even for researchers. As Web data and search engines also have limited access to researchers, we chose to use Twitter data.

However, selecting tweets that could be associated with actual uptake of drugs to determine which drugs are widely used and to derive insight from corresponding tweet topics is still a challenge.

### Related Work

#### Medical Entity Estimates From Twitter

To obtain widely used drugs (or any other medical entity) from tweets, one needs to count the tweets with mentions of drugs (medical entities) and rank their frequencies. In previous studies, Twitter has been used to estimate the extent to which medical entities (drugs, diseases, symptoms) mentioned in tweets have been experienced or will be experienced by aggregating counts and then finding correlations with official surveillance data. For example, flu epidemics have been predicted in previous studies [11,23]. Correlations between flu or disease mentions in Twitter data and official flu case data have been found as well [12,18,19]. With regard to drugs, Twitter has been effectively leveraged to study prescription drug abuse [13], to track usage of illicit drugs [14], and to find a correlation between flu vaccine sentiment tweets and official vaccination rates according to geography [24].

The presence of medical entities in tweets does not necessarily signify that people have the illness, are using the pharmacological substances (drugs), or are exhibiting certain symptoms. Therefore, to obtain frequency of drug use from tweets, it is best to first identify which tweets indicate that tweeters have consumed the drugs mentioned in their tweets.

Several previous studies have paid attention to the identification of actual experiences of medical entities from other mentions. This includes work by Aslam et al [25] and Ji et al [26] who sought first to differentiate between *valid* and *invalid* tweets that expressed experiences with the flu before proceeding with further analyses. Weeg et al [27] found that the correlation between population disease prevalence and disease mentions in tweets increased from .113 to .208 (*P*<.001) when only disease name mentions that refer to actual diseases were taken into consideration. Using a somewhat similar approach, Alvaro et al [28] identified tweets that described firsthand experiences of prescription drugs, which were then used to gather evidence about adverse drug reactions.

Due to the large volumes of Twitter data, an effective way to separate the class of tweeters with experience with medical entities from the nonexperience class is by using machine learning (ML) techniques. However, researchers are still striving to improve the performance of the ML classifiers used for classifying tweets into those respective classes.

#### Topic Extraction From Twitter

To obtain insight from tweeters who have consumed these drugs, we sought to extract categories or topics from their respective tweets. Twitter data have been used in previous studies to extract categories or topics about health-related issues for various purposes. We have seen Twitter being used to investigate topics surrounding antibiotic prescription drugs [15] to find emerging trends for tweets related to electronic cigarettes [16], to detect topics at the peak of a disease related to official reports [29], and to find trending topics from preselected health-related keywords [17].

However, most of these studies conducted their topic analysis by classifying their tweets into predefined health categories [15,16] instead of obtaining these categories automatically. Those approaches miss opportunities to discover hidden and new topics. Additionally, the discovered topics were extracted from all the tweets that mentioned the medical entities [29]. However, only the tweets that expressed experiences with medical entities should be included in the topic extraction [17].

#### Influenza Drug Uptake Surveys

Uptake of drugs to treat or prevent influenza infections is an effective measure to mitigate the impact of influenza outbreaks. Consequently, there is a need to understand which factors contribute to the increase or decrease of drug uptake. With that information, public health agencies can take appropriate action against factors that may decrease drug uptake and promote factors that may increase drugs uptake.

Among the factors that deter uptake of influenza drugs (especially vaccines) are fear of needles, pain, and distress resulting from vaccination [30-32].

The logistics of vaccination processes that include the locations of vaccination centers and waiting time are among the factors that influence people to receive an influenza vaccine [32-34]. Some people tend to prefer traditional vaccination locations (hospitals, clinics, and doctor’s office) [32], whereas others prefer nontraditional locations (pharmacies, workplaces, and schools) [33,34]. Short line-ups and wait times help facilitate influenza vaccine uptake [34].

There have been concerns about the safety of the influenza drugs with respect to people with allergies [35] and pregnant women [36-39], which negatively affect the uptake of drugs.

Demographic factors, including age and level of education have been found to contribute to the uptake of influenza vaccines in which younger and less-educated adults are more hesitant to take drugs than other groups [40].

Public health agencies and pharmaceutical companies, as well as doctors or medical professionals [41,42] and parents [42] are also positive influences on drug uptake.

It has also been observed that some people prefer natural remedies to conventional drugs for influenza treatment or prevention [22]. However, natural remedies should be used to supplement and not replace tested influenza drugs.

The findings of these studies provide useful public health information for controlling the severity of seasonal influenza outbreaks through drug uptake. However, the methods for data collection used in most of these studies, that is, face-to-face interviews [30-32,37,41], written questionnaires [34,38], telephone surveys [33,42], secondary data databases [36], and Web page hits [22] have many drawbacks that may adversely affect the quality of the findings.

The cost for conducting these survey studies is one of the limitations. For example, a typical clinic-based survey (interview) for one participant in the United States costs US $23.51 compared with US $14.63 for a social media survey [43]. In another study [44], the cost of a telephone survey per sample (US $3.98) was higher compared with Web-based surveys (US $0.71). These high costs may hinder collection of data from large sample populations.

For public health agencies to take immediate and effective action, the findings of these studies should be released as soon as possible. However, these conventional methods are subject to delays with regard to data collection and may provide results later than they are needed to make informed decisions [45].

To employ effective strategies against seasonal influenza outbreaks, research results that reflect the actual situation in the population being studied should be used. However, because of the nature of the interactions between researchers and respondents in conventional or traditional methods, these studies are affected by social desirability bias in which respondents tend to provide responses that seem favorable [46]. This trend may lead to incorrect analysis results and ultimately cause public health agencies to fail to take appropriate action in locations where it is required.

Additionally, the size of study populations, which are often scattered in disparate geographical regions, can limit coverage in these studies. It is very costly to conduct studies with a population size that can be generalized to larger populations such as regions and countries.

In summary, existing approaches have two main limitations. First, the performance of ML classifiers used for identifying experience or personal tweets need improvements. These approaches mainly used n-gram bag-of-words or characters as ML features. These features suffer from the curse of dimensionality because the total dimension of each tweet’s text is equal to the vocabulary size, which can overfit the models. Additionally, these features do not consider semantic relations between words, which can result in poor performances in some cases.

Second, traditional surveys for finding insights about influenza drugs suffer from limitations associated with cost, timelessness in reporting, coverage, and bias.

### Objectives

To achieve our research aim, we set the following objectives as our guidelines:

The first objective was to provide a methodological extension for efficiently extracting more widely used drugs during seasonal influenza using tweets with evidence for consumption of drugs. We focused on providing an improved ML classifier that could identify tweets indicating uptake of drugs from others. We hypothesized that using a dependency words structure (introduced in the Methods section) of the tweets as our features would improve performance in classification.

Our second objective was to extract topics from tweets mentioning each of the widely consumed drugs and to infer how the insights provided by these topics can enhance seasonal influenza surveillance in lieu of relying on traditional surveillance. From this perspective, we focused on automatically (without predefined categories) finding fine-tuned topics (extracted separately from tweets about each drug). We hypothesized that topics of widely consumed drugs could be used to enhance surveillance of seasonal influenza. Additionally, the tweets from these topics were the ones that signified actual consumption of drugs.

The contribution or significance of the research is two-fold in the following ways:

First, we proposed a new enhanced classification method to identify whether a tweet indicates someone has consumed or intended to consume a drug or not. This enhanced classification method guarantees results that reflect the actual situation in the population being studied when searching for widely used drugs and in subsequent analyses (topic extraction).

Second, a topic extraction–based method was applied to analyze the hot topics in tweets that can help public health stakeholders enhance seasonal influenza surveillance and intervention measures in terms of drug administration and consumption. Instead of using traditional or conventional survey methods, the topic extraction–based method can directly extract topics from tweets of people who have consumed these drugs. This sort of approach guarantees cost-effective, fast, and high coverage results for public health stakeholders to take action if needed.

To the best of our knowledge, this paper is the first to use a topic extraction–based method to retrieve insights about seasonal influenza drugs using Twitter data. The retrieved topics can highlight enabling and deterrent factors to drug uptake.

We evaluated our ML classifier by comparing it with a 1-gram term frequency (TF)–based classifier and a clue (keyword or lexicon)-based classifier. These benchmarks were chosen because they have been extensively used for separating tweets of individuals with actual experience (personal or valid) from tweets made by others [25,26,28,47-50]. For the extracted topics, we examined how the findings support the results of previous studies that used conventional or traditional surveillance methods.

## Methods

### Overview

**Figure 1:**
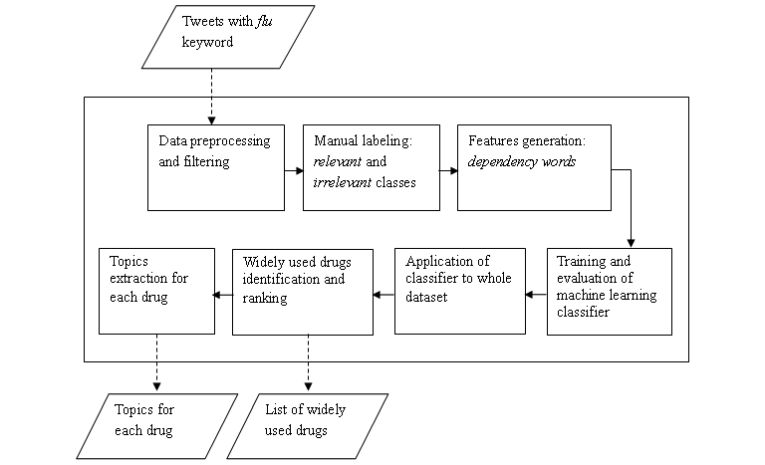
An overview of the proposed method.

The overview of the proposed method is summarized in Figure 1. The tweets are first preprocessed and filtered to obtain tweets with drug mentions only. Then, a random sample of these tweets is manually annotated as relevant or irrelevant with respect to actual drug uptake evidence. Next, features to be used by our classifier are generated. The classifier is trained, evaluated, and applied to the whole dataset to identify tweets indicative of the consumption of drugs (relevant tweets). From these tweets, we derive (1) a ranked list of widely used drugs and (2) topics from tweets mentioning each of these widely used drugs. A detailed description and implementation of these steps are provided in the following sections.

**Table 1:** Samples of relevant and irrelevant tweets with mentions of drugs (drugs italicized).

Category	Tweet text
Relevant	*Nyquil* cold&ampFlu thanks for my life back!
	Got my *flu shot*. Ughhh.
	I got the *flu vaccine* that means I am gunna get sicky.
Irrelevant	We have *flu shots* In! Make Your Appointment Today 642-###
	He got a song about some damn *Thera flu* lol
	So I either have the flu or a mild case west nile virus! *FML*!!

### Data

We used historical Twitter data for the 2012-2013 influenza season collected from August 31, 2012 to March 04, 2013 within 30 cities in the United States using the Twitter application programming interface (API) and the keyword *flu*. This approach ensured that the analyzed drugs were related to seasonal flu outbreaks. Only tweets written in the English language were considered, and in total there were 459,043 tweets included in the analysis.

Post collection filtering was done to remove tweets that contained the keyword *flu* but were unrelated to the flu disease. For example, some tweets contained the phrase *stomach flu*, which implies an intestinal infection. Additionally, tweets that had the keyword *flu* as part of the Twitter username or handle or Twitter hashtag (such as @this_is_flu or #flu_camp, respectively) were also removed from our dataset. Because the majority of retweets and tweets with URLs do not usually express actual experiences of health-related issues [25], they were also removed from the dataset.

To identify the tweets that mentioned drugs, the unified medical language system (UMLS)-MetaMap [51] developed at the US National Library of Medicine was used. The semantic type of each token or word in a tweet was first identified by the UMLS-MetaMap. If the word was classified into one of the following three UMLS semantic types: immunological factor, clinical drug, or pharmacological substance (which are the semantic types for preventive or treatment drugs), then the corresponding tweet was kept for subsequent analyses.

### Manual Annotation of Tweets

Since we wanted to train an ML classifier that could identify tweets indicating uptake of drugs, we first manually labeled a random sample of the tweets in two classes, including the *relevant* class if a tweet indicated actual consumption of drugs and the *irrelevant* class for other topics. These manually labeled data were later used to train and test the classifier. Table 1 has some examples of relevant and irrelevant tweet labeling.

Two annotators were tasked with categorizing 5000 tweets into the two classes (relevant and irrelevant). The interrater agreement between the 2 annotators measured using Cohen kappa [52] was .91. A third annotator was recruited to decide on the disagreements between the 2 annotators through majority votes.

### Machine Learning Classifier Features

The proposed classifier used dependency words as features that were obtained as follows: We parsed each tweet to obtain the dependency structure of words, and then we identified which words in the tweet had dependency relations with a drug mentioned in the tweet; we only used these words as our features. Our approach was peculiar in the sense that we did not use the dependency grammar categories (eg, nsbuj, dobj, and conj) of the words in the tweets as features. We named this feature *dependency words*.

TF-inverse document frequency (IDF) basically represents each text document (in this case, a tweet’s dependency words) as a vector of terms or words with each component of the vector corresponding to each term in the corpus and has a weight or count associated with it [52]. The TF part measures how frequently a term occurs in a document, whereas the IDF part weights the most frequent terms while scaling up the rare ones. For corpus terms that are not in the document, their weights are zero. The terms can have any n-gram (n=1, 2...) size. However, Cole-Lewis et al [16] determined that using 1-gram instead of other n-grams to classify tweets into relevant and irrelevant categories led to the best performance. Since we intended to implement our model with a support vector machine (SVM) algorithm that also scales the terms, we used TF only. Thus, a 1-gram TF of dependency words was adopted in this study to categorize tweets into relevant or irrelevant types.

The Stanford parser (Stanford University) [53] was used to find dependency words, whereas during TF feature construction, we used the Stanford CoreNLP [54] to reduce the dependency words into their lemma forms.

The following example shows the dependency words extracted from a sentence in a tweet:

Original sentence:
*Just got my Nyquil, ready for bed*.

Results:
*my*,
*got*, and
*ready*

From the example, the drug mentioned in the tweet is Nyquil, which has grammatical dependency relations with the words *my*, *got*, and *ready*.

### Classifier Training, Evaluation, and Application

We used the SVM with a linear kernel function as the ML algorithm, which was implemented using LIBSVM from the National Taiwan University [55]. To obtain a trained SVM model that can provide optimum classification results, there are two parameters that must be tuned, namely C and gamma. We applied a grid search method on C and gamma using 10-fold cross validation to test several pairs of C and gamma, and the pair that provided the best 10-fold cross-validation performance for the training set was chosen. With these parameters, the classifier was then trained with 3400 labeled data, and its performance was evaluated using 1600 test data in terms of precision, recall, and F_1_ score. To ascertain the classifier’s ability to avoid overfitting, the classifier was tested by evaluating its performance on the test dataset that was not used for training. This test dataset was assumed to approximate the typical unseen data the classifier would encounter in the future.

Our training set had a total of 701 features (dependency words). We tested whether we could achieve the same or better performance with fewer features through feature selection (20%, 40%, 60%, and 80%), but we could not get better performance with less than 100% of the features. Therefore, all of the features were used.

To evaluate the performance of our proposed ML classifier, we compared it with two benchmark classifiers, including a 1-gram TF-based classifier used in prior studies [25,28,47-49] and a lexicon (keyword or clue)-based classifier as used in another previous study [26]. The 1-gram TF classifier was constructed by using all of the words in each tweet after removing all of the named entities (eg, person, location, organization, numerical, and temporal) and stop words, which are considered to have little or no contribution to document classification. The lexicon-based classifier was constructed by considering the presence of subjectivity words, news keywords, and profanity words. A tweet was considered to indicate drug experience (relevant) if it contained at least 3 strong subjective words and 3 weak subjective words. In contrast, a tweet was considered to be irrelevant if it contained news keywords, and there were no profane words.

The optimum-trained model was then applied to the whole corpus of tweets to find all relevant tweets, that is, tweets indicating consumption of drugs. We counted the drugs mentioned in these tweets and ranked them to identify widely consumed drugs.

### Topic Extraction for Tweets Mentioning Drugs

Topic models are based on a concept that documents are mixtures of topics in which a topic is a probability distribution for words [56] *.* In other words, a topic refers to a group of words that frequently occur together and can form meaningful and interpretable themes. Latent Dirichlet allocation (LDA) is one of the simplest topic models and is widely used in Web text mining.

LDA is a generative model for topic modeling that can automatically discover hidden topics from a collection of text documents represented as a bag-of-words [57]. Each document is regarded as a mixture of several topics, and a topic is a distribution of words. For a collection of text documents, the LDA model can generate a certain number of topics. By understanding the topic distributions among text documents and the word distributions among topics, unknown or hidden information in the text can be retrieved.

The goal of topic modeling was to separately discover hidden topics from a collection of relevant tweets for each widely used drug. For the tweets of each drug, we applied the LDA topic modeling method to find topics. The LDA method was implemented by MALLET (University of Massachusetts Amherst) [58].

The number of topics retrieved for tweets about each drug was varied using an optimum topic number test as suggested by a previous method [59]. We applied the LDA topic model to the documents (tweets) with a randomly specified number of topics and observed the per-document topic distributions results. If the per-document topic distributions of all documents were dominated by a few topics, then the number of topics was increased and vice versa, until an optimal balance was found. The only parameter or value we specified was the number of topics. Other LDA parameters were tuned automatically from their default values (alpha=5.0 and beta=.01) based on the specified number of topics and based on the number of words in the tweets. Detailed information on our implementation of the LDA-based topic model for tweets of widely consumed drugs is provided in Multimedia Appendix 1.

The results of this LDA topic model included per-document topic distributions, a set of topics, and word-to-topic assignments for each word in the corpus. However, the data that were most interesting to us were the extracted topics.

## Results

### Relevant Tweets With Drug Mentions

After the initial preprocessing and filtering of tweets to remove retweets, as well tweets with URLs and tweets with the keyword *flu* that did not imply the flu disease, we had 220,375 tweets remaining. The number of tweets with drug mentions was 50,828 after filtering with MetaMap software. The number of relevant tweets that indicated actual uptake of drugs after applying our classifier was 40,428. These 40,428 tweets produced 6232-word vocabulary size (ie, number of dependency words).

### Classifier Performance Evaluation

The performance of our SVM classifier, when applied to the test set measured in terms of precision, recall, and the F_1_ score is shown in Table 2. The gamma and C parameter values for the SVM classifier that provide the optimum performance were .008 and 8, respectively, after a thorough grid search. Our proposed model, which was 1-gram TF of dependency words, outperformed the model constructed using 1-gram TF and the lexicon-based model as depicted in Table 2.

The test dataset was divided into 30 parts, and a statistical significance test of our classifier over the two benchmark classifiers was conducted to check whether the proposed method outperformed the two baseline methods significantly. The results showed that the differences in the F_1_ score between our classifier and the two benchmarks were statistically significant (ie, *P*<.001 with the lexicon-based model and *P*=.048 with the 1-gram TF model).

**Table 2:** Performance of the classifiers using lexicon-based, 1-gram term frequency (TF), and dependency word features.

Classifier features	Precision	Recall	F_1_ score
Lexicon-based (benchmark 1)	0.52	0.91	0.66
1-gram TF^a^ (benchmark 2)	0.73	0.88	0.79
Dependency words (our approach)	0.77	0.90	0.82

^a^TF: term frequency.

**Table 3:** Widely used drugs retrieved from relevant tweets (N=40,428).

Drugs	Tweets count, n (%)
Influenza virus vaccines	31,111 (76.95)
Theraflu	1267 (3.13)
Vitamins	439 (1.09)
NyQuil	354 (0.88)
Acetaminophen	270 (0.67)
Oseltamivir	162 (0.40)
DayQuil	75 (0.20)

**Table 4:** Relevant topics retrieved from tweets mentioning influenza virus vaccines (only interpretable topic compositions are listed).

Topic number^a^	Topic compositions
1	mom, today, needles, doctor, nurse, gave, give, told, dad, baby, big, shot, giving, making, wanted, lady, sh*t, f*ck
2	reaction, hoping, sick, kids, allergic, eggs, made, egg, chicken, allergy, medicine, tea
3	flu, season, people, year, virus, immune, system, shot, epidemic, strain, stay, healthy, spreading, protect, remember
4	influenza, pregnant, risk, vaccination, national, recommend, women, immunity, free, safe
5	arm, sore, today, hurts, hurt, yesterday, damn, left, feels, side, bad, feel, stupid, throat, pain, ouch, killing, feeling, hurting
6	waiting, cvs, line, walgreens, pharmacy, free, wait, office, give, long, gave, spray, clinic, nasal, giving, people
7	sick, hate, shots, f*ck, sh*t, flu, needles, damn, today, nervous
8	work, office, day, today, free, morning, tomorrow, doctors, doctor, shot, good, school

^a^Corresponding interpretations of these topics are in Table 5.

### Widely Used Drugs

Table 3 shows the counts for the selected widely used drugs and their percentages. We only considered drugs with tweet count percentages that were at least 0.20%. We excluded some *drugs* that the MetaMap software identified as drugs, but the tweets did not imply drug use. We did a manual analysis of these *drugs* by going through some of the tweets they came from. For example, *RID* in many of the tweets was used to mean *to clear or to free*, but it was identified as a drug because it could also mean pyrethrins or piperonyl in other contexts. This outcome occurred because the syntactic structure of tweets with these *drugs* was similar to tweets with mentions of true drugs, which made it difficult for the classifier to detect this ambiguity automatically.

Since we wanted to obtain frequencies or counts of drug uptake only, we did not include water and disinfectant products in the table, which were also identified as widely used drugs.

Although some drugs were identified separately by the MetaMap software, they are actually the same. Therefore, we grouped these drugs into one category for the sake of simplicity. For example, flu shots, flu vaccines, flu vaccine, vaccines, and vaccine were combined and presented as one category or class called influenza virus vaccines, and vitamins and vitamin C were grouped into vitamins.

### Topics for Drugs-Mentioning Tweets

The optimum number of topics retrieved for each *relevant* drug was as follows: Influenza virus vaccines (10), Theraflu (5), DayQuil and NyQuil (5), vitamins (5), acetaminophen (3), and oseltamivir (3).

Table 4 shows topics of tweets with influenza virus vaccine mentions. Multimedia Appendix 2 has the full results of topic extraction for all drugs, which includes the number of topics, topic compositions, and LDA parameters.

In Table 5, we presented interpretations of the topics (clusters of frequently occurring words) for the drugs listed in Table 3. These interpretations express the meanings that the topics convey.

**Table 5:** Interpretations of topics retrieved for each drug.

Drugs	Topic interpretations
Influenza virus vaccines^a^	Vaccination proponents, vaccination allergic reaction, vaccination reminders, vaccination pregnancy risk, vaccination pain and distress, vaccination queues concerns, vaccination fear, vaccination places
Theraflu	Natural flu remedies uptake (chicken soup, hot drinks)
DayQuil or NyQuil	Drug uptake time (morning, night)
Vitamins	Flu preparedness through vitamins intake
Acetaminophen	Symptoms; Natural flu remedies uptake (soup, tea, orange juice)
Oseltamivir	Prescription of drug

^a^Correspond to topic numbers in Table 4.

## Discussion

### Principal Findings and Comparison With Previous Studies

#### The Classification Method

When compared with the two benchmarks, our proposed method showed performance (F_1_ score) improvement for classifying tweets with mentions of drugs into *relevant* and *irrelevant* categories. This improvement was significant compared with both the 1-gram TF classifier (*P*=.048) and the lexicon-based classifier (*P*<.001).

The ML classification intention was designed to find which tweets indicate that a tweeter has consumed or intends to consume the drugs mentioned in their tweets. We used dependency parsing to first find which words in the tweets were involved in the binary grammatical relations with the drugs mentioned in the tweets, and then by using these words as our ML classifier features, we achieved better performance. This result occurred because these words (features) are closely related or aligned to the drugs and serve as better distinguishing features than using all words (1-gram TF) or even had much better results than using lexicon-based methods. Additionally, as dependency words were used only as features, the approach is not affected by dimensionality, and there is no possibility of model overfitting.

The two benchmarks were chosen because of the fact that they have been extensively used to identify *valid or relevant or personal* and *invalid or irrelevant or nonpersonal* tweets regarding flu experience, which is similar to the procedures used in prior studies [25,26,28,47-50].

#### Widely Used Drugs and Corresponding Topics

In this study, we defined *relevant* tweets as the ones that were composed by people who express their or another person’s consumption of drugs. Out of 50,828 tweets that mentioned drugs, 40,428 (79.54%) were relevant tweets. This result implied that people discuss health-related conditions and actions on Twitter for both themselves and other people. This outcome is consistent with the work of Yin et al [10] who investigated whether Twitter users disclose health statuses (either their status or the status of other people).

The research estimated the proportions of different drugs used during the flu season. The results showed that widely used drugs included influenza virus vaccines 76.95% (31,111/40,428). Previous studies also investigated the uptake of influenza drugs, especially vaccines [60,61]. However, these studies mostly relied on surveys or interviews that were prone to social desirability bias, delays, and were very costly. Our approach leveraged tweets to obtain these proportions quickly and at a low cost, which can efficiently reflect the actual drug uptake.

Furthermore, in this research, we found that the topics of tweets mentioning the drugs varied depending on the types of drugs that were discussed. These topics could not be easily and accurately found using normal search queries [15] or traditional survey methods because of their limited scope. Combining mentions of widely used drugs together with trending topics can be beneficial to various public health stakeholders for controlling flu epidemics.

Regarding influenza virus vaccines, we observed that during flu seasons, people who were vaccinated tended to remind others to do the same to protect themselves from the flu and stay healthy (Table 4, topic 3: flu, season, people, year, virus, immune, system, shot, epidemic, strain, stay, healthy, spreading, protect, remember). This is a positive sign because lack of reminders has been found to be one of the barriers to vaccination among adults [62]. The following tweet shows how people who get vaccinated urge others to do so:

Guys protect yourself this flu season. Just got my mandatory flu shot. #HealthyPeople

Additionally, apart from medical personnel, we noticed that people got their flu vaccines because of the persuasion or presence of their parents (Table 4, topic 1: mom, today, doctor, nurse, gave, give, told, dad, baby, big, shot, giving, making, wanted, lady, sh*t, f*ck). The presence of *curse words* and *needle* indicates pain resulting from vaccine injections, whereas the words *doctors, nurse, moms,* and *dads* indicate that vaccines were given or offered in the presence of these people or these people influenced the vaccination process. This result provides information on which cohort to target for sensitization campaigns to have a more positive impact on flu medication uptake, especially preventive drugs such as flu vaccines or shots. According to Giese et al [63], individuals are likely to be vaccinated if their doctors or medical staff have recommended the vaccine. Our topics analysis has discovered another potential group (parents) that can also facilitate convincing more people to get vaccinated because they were involved in the vaccination process. We expected parents to make vaccination decisions for young children only [64,65]. However, as Twitter users are mainly adults, it appears that parents can influence adults too:

...my moms making me get the flu shot ):

Moms making me get the flu shot. I f****ng hate needles.

Idk my dad wants me to get a flu shot he knows I hate needles.

Additionally, the extracted topics indicated that people got or had intended to get their vaccines at places where they were spending most of their time during the day and where the drugs were offered or given for free (Table 4, topic 8: work, office, day, today, free, morning, tomorrow, doctors, doctor, shot, good, school). The presence of topic words such as *work*, *office*, *school*, and *free* means that vaccines were given at those places (mostly for free). The finding can imply that offering drugs at these places can be an effective decision because of the peer pressure effect, rather than offering these drugs at health facilities only. This result correlated with the finding that people who receive vaccines are individuals who are in contact with many others during their daily activities [66]:

My office is giving out free flu shots I think I will go, last year my insurance did not cover my flu shot.

The topic analysis also showed that people who got or had intended to get their vaccines were concerned with waiting for a long time in vaccination queues (Table 4, topic 6: waiting, cvs, line, walgreens, pharmacy, free, wait, office, give, long, gave, spray, clinic, nasal, giving, people). The words *line*, *long*, and *waiting* meant that waiting lines were long, or people waited for a long time in lines. The words *cvs*, *walgreens*, *pharmacy*, *office*, and *clinic* are vaccination locations. These results imply that time spent waiting on long lines at vaccination centers was a concern for many people, and measures need to be taken to address that concern:

2 hours in line at cvs for a flu shot #sickofthissh*t.

Additionally, the aftermath of pain and distress from vaccines were among the concerns of many people (Table 4, topic 5: arm, sore, today, hurts, hurt, yesterday, damn, left, feels, side, bad, feel, stupid, throat, pain, ouch, killing, feeling, hurting). The presence of words such as *hurts*, *hurt*, *hurting*, *sore*, *damn*, *ouch*, and *pain* indicates pain, whereas the words *arm* and *left* indicate the body part affected by the pain. This finding can help health administration to design better strategies for offering these drugs and to avoid discouraging intended recipients in the future. For example, the 5P (procedural, physical, pharmacologic, psychological, and process) pain management intervention strategies [67,68] can be employed to ease pain and distress from drugs. The following tweets indicate the pain and distress users tend to associate with vaccine use:

I got the flu shot today and my arm STILL HURTS I’m all sore.

This FLU SHOT after effect is KILLING ME. My arm so sore and in pain OMG.

Vaccination needle fear was also among the themes that emerged from the tweeters (Table 4, topic 7: sick, hate, shots, f*ck, sh*t, flu, needles, damn, today, nervous). This means sensitization needs to be conducted to ensure that more people overcome their fear and partake in vaccination. The following tweet indicates the fear of needles:

Getting a flu shot. So nervous. Hate shots.

Tweeters with egg or chicken allergies raised concerns about whether the vaccines would cause an allergic reaction (Table 4, topic 2: reaction, hoping, sick, kids, allergic, eggs, made, egg, chicken, allergy, medicine, tea). This outcome implies proper education should be given to avoid scaring away people with egg or chicken allergies or other allergies:

@Twitterhandle That’s good to know. Both me and my little girl have egg allergy and got no ill effects from the flu shots.

There were also themes showing concerns about the risk of vaccination to pregnant women (Table 4, topic 4: influenza, pregnant, risk, vaccination, national, recommend, women, immunity, free, safe). This finding means people were concerned with the efficacy and safety of the vaccines, which is similar to the findings of another study [69] and suggests there should be strategies to improve vaccination uptake among pregnant women.

1st getting my flu shot. Is it safe to get a flu shot during pregnancy? Yes. Better safe than sorry.

Some topics for Theraflu and acetaminophen were related to how people who consumed these drugs also tended to use natural or home flu remedies (Multimedia Appendix 2, table MA3, topic 1: soup, chicken, noodle, juice, orange, care, flu, work, sleepy, warm, easier, food, drinkin, spicy, vodka) and (Multimedia Appendix 2, table MA5: fever, throat, juice, recommend, drowsy, soup, temp, symptom, strep, spray, orange, tea). However, there is a need to provide awareness to people so that they use these home remedies only as early interventions or in conjunction with clinically prescribed drugs for effective treatment of the flu [70]. The following tweets indicate that some people use drugs such as Theraflu and acetaminophen in conjunction with home remedies:

Chicken noodle soup and thera flu for dinner...mmm.

@Twitterhandle drink some green tea and chicken soup and take theraflu and get rest#lets earn two.

For the other drugs (except for influenza vaccines, Theraflu, and acetaminophen), the observable interpretable topics were related to preparedness against flu, symptoms, and prescriptions, as well as the use of the drugs to prevent or combat the flu.

Overall, in this research, we went the extra mile by ensuring that the topics about drugs reflected actual experiences of medical entities or drugs and that not all mentions of drugs were included, as they were in prior studies [17,29]. Additionally, these topics were supported by findings from several studies that are related to influenza surveillance but used traditional survey methods.

### Limitations and Future Work

The study succeeded in improving the performance of the classifier when compared with the benchmarks and managed to find important themes about the consumed drugs. However, the study was limited by challenges such as misspellings, abbreviations, and slang language, which are common issues facing researchers attempting to analyze social media text. As most tweets were composed by individuals with no medical backgrounds, the tweets had lots of misspellings when mentioning drugs. Additionally, because of space restrictions and the informal nature of Twitter conversations, people often opted to use abbreviations and slang when exchanging messages. In this study, we automatically extracted treatment drugs using MetaMap software, which recognized only standard medical names and left out some tweets with drug mentions. This might have caused the number of tweets with a specific drug to be reduced and made it hard to find all of the meaningful topics. To overcome these challenges, future research studies should consider using MetaMap software combined with appropriate misspelling identification and correction methods. Additionally, abbreviations and slang should be expanded to their full forms and interpreted using appropriate lexica.

Additionally, we did not explore correlations of the uptake of the drugs between tweets and official statistics. We also did not conduct analyses in smaller, more specific geographical locations (such as cities). Instead, we considered the country as a whole. Future research studies can conduct analyses at the city level and find correlations between results from tweets and official data.

### Conclusions

As the number of users and the sharing of health information on Twitter increase, Twitter has turned out to be a potential data source for public health research. It can help to investigate the uptake of various drugs and perceptions of users toward those drugs during seasonal flu outbreaks. Analyzing these massive datasets requires efficient methods that can identify emerging trends in the uptake and administration of drugs. Using ML techniques, this study proposed a methodological extension for efficiently extracting more widely used drugs during seasonal influenza using tweets confirming the consumption of drugs during seasonal flu outbreaks from a pool of tweets with mentions of drugs. Emerging topics of each confirmed tweet about drugs were extracted, which provided hidden information conveyed by people who have consumed these drugs. Mainly, these insights included encouraging or discouraging factors for influenza drug uptake. This information was obtained automatically rather than being obtained by conventional methods that have many shortcomings. Therefore, public health entities and other stakeholders can make full use of this efficiently obtained information to devise efficient and effective strategies for influenza epidemic surveillance.

## Appendix

Multimedia Appendix 1 Latent Dirichlet allocation (LDA) topic model and implementation.

Multimedia Appendix 2 Topics for tweets with drug mentions.

## Abbreviations

API: application programming interface
ILI: influenza-like illness
LDA: latent Dirichlet allocation
ML: machine learning
SVM: support vector machine
TF: term frequency
TF-IDF: term frequency-inverse document frequency
UMLS: unified medical language system
