# Supplementary material for: Enhancing Seasonal Influenza Surveillance: Topic Analysis of Widely Used Medicinal Drugs Using Twitter Data
Source: J Med Internet Res. 2017 Sep 12;19(9):e315. doi: 10.2196/jmir.7393 (PMC5617904; doi:10.2196/jmir.7393)
Supplement: Multimedia Appendix 1 [file jmir_v19i9e315_app1.pdf]

Multimedia Appendix 1: LDA topic model and implementation

LDA Model

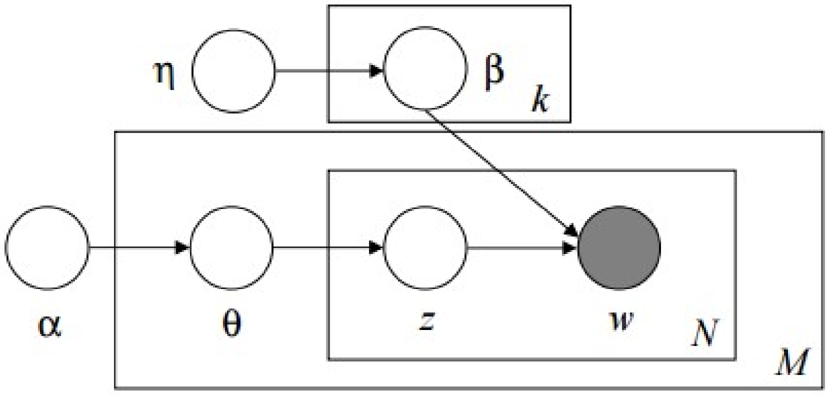

Figure 1. LDA Model

The generative process is described as follows. [1]

- Draw topic distribution θ_i for all posts I in the collection M by θ_iDirichlet(α). The Dirichlet(α) is the Dirichlet distribution with parameter α.
- Draw word distribution for all the topics β_k in the collection of k by β_kDirichlet(η).
- Finally, for each of the word position i, j:
    - choose a topic z_{ij} Multinomial(θ_i)
    - choose a word w_{ij} Multinomial(βz_{ij}).

In our implementation, for each drug, the dataset was a collection of M drug mentioning tweets sharing same k topics. A drug mentioning tweet was considered to consist of several possible topics, such as symptoms, vaccination fear, vaccination pain, and distress. The topics were hidden in the model; thus we did not know them beforehand. For each tweet, topic distribution can be different. We denoted θ_i as the topic distribution over tweet i. This θ_i was different for different tweets. The probability of choosing a specific topic on tweet i, word position j (e.g., vaccination fear on the first-word position of the tweet), was denoted as P ($z_{ij}|\theta_i$). Topics are related to words. The vaccination pain and distress topic has a closer relationship with the words "arm", "sore", "hurt", "damn" whereas the vaccination fear discussion topic has a closer relationship with the words "hate", "shots", "f*ck", "sh*t", "flu", "needles", "damn", "nervous". Therefore, different topics had different word distributions over the whole vocabulary. We denoted β_k as the word distribution for topic k. Therefore, a specific word w_{ij} on post i, position j had the probability P ($w_{ij}|\beta z_{ij}$).

We can only observe words from the model as shown in figure 1 above that is why only the "words" circle is shaded. Our goal was to identify hidden topics and topic-related keywords. By

feeding the collection of tweets into the model, we could estimate the parameters θ_i and β_k by using maximum likelihood estimation. Although θ_i was used to indicate topics for a given tweet, β_k was used to describe the meaning of topics based on the distribution of words.

Implementation using software – MALLET

The goal of topic modeling was to discover hidden topics from a collection of relevant tweets of each widely used drug separately. For each drug's tweets, we applied LDA topic modeling method to find its topics. The LDA method was implemented by MALLET [2].

The number of topics retrieved for tweets of each drug was varied using optimum topic number test as suggested in [3]. We applied the LDA topic model on the documents (tweets) with a randomly specified number of topics and observed the per-document topic distributions results. If the per-document topic distributions of all documents were dominated by a few topics then the number of topics were increased, and vice versa until an optimal balance was found. The only parameter/value we specified was the number of topics. Other LDA parameters were tuned automatically from their default values (alpha = 5.0 and beta = 0.01) based on the specified number of topics and content of the tweets.

A typical situation that we encountered that required us to increase the number of topics is shown in the per-document topic distributions table below (Table 1). The table shows selected 12 tweets and their topic distributions when the number of topics was set to 2. As it can be seen, the majority of tweets ended up or contained topic 2, which prompted us to increase the number of topics.

Table 1. Per-document topic distributions

Tweet/document number	Topic 1	Topic 2
1	0.65	0.35
2	0.04	0.97
3	0.23	0.77
4	0.1	0.9
5	0.92	0.08
6	0.08	0.92
7	0.76	0.24
8	0.13	0.88
9	0.12	0.88
10	0.11	0.89
11	0.17	0.83
12	0.09	0.91

References

1. Zhan Y, Liu R, Li Q, Leischow SJ, Zeng DD. Identifying Topics for E-Cigarette User-Generated Contents: A Case Study From Multiple Social Media Platforms. J Med Internet Res, 2017. **19**(1): p. e24. DOI: 10.2196/jmir.5780. PMID: 28108428.
2. McCallum AK. MALLET: A Machine Learning for Language Toolkit. 2002. URL: http://mallet.cs.umass.edu [accessed: 2016, 21 August]; Archived at: http://www.webcitation.org/6m3SO8CIp
3. Graham S, Weingart S, Milligan I. Getting started with Topic Modeling and MALLET. The Programming Historian, 2012. **2**: p. 12.
