# Supplementary material for: Enhancing Seasonal Influenza Surveillance: Topic Analysis of Widely Used Medicinal Drugs Using Twitter Data
Source: J Med Internet Res. 2017 Sep 12;19(9):e315. doi: 10.2196/jmir.7393 (PMC5617904; doi:10.2196/jmir.7393)
Supplement: Multimedia Appendix 2 [file jmir_v19i9e315_app2.pdf]

Multimedia Appendix 2. Topics for tweets with drug mentions (raw).

^{w} means the topic with the highest weight in the group

Table MA1. Dayquil/Nyquil topics (Beta = 0.34)

**Topic Number**	**Alpha**	**Topic Compositions**	**Interpretation**
1	0.16	feeling, living, working, knock, shot, made, stay, horrible, cold/flu, call, nasty, finally, thankful, celebrating, drinking, weak, prefer, pass, nose, times	unknown
2^{w}	0.99	flu, cold, sleep, time, sick, night, day, feel, symptoms, bed, soup,	uptake time
3	0.15	eat, cycle, throat, pretty, work, due, medication, popped, z-pack, clears, coma, idea, hoping, barely, dosed, discontinued	unknown
4	0.41	flu, season, cold, top, learn, form, brittany, cough, aids, drops, works, knocked, tonight, watching, love, tissues, tea	unknown
5	0.13	shots, cold&flu, half, party, hard, sum, big, boss, talk, planned, poorly, capsule, grab, end, man, sounds, feeling, counter, gallon, marijuana	unknown

Table MA2. Influenza virus vaccines topics (Beta = 0.05)

**Topic Number**	**Alpha**	**Topic Compositions**	**Interpretation**
1	0.10	mom, today, needles, doctor, nurse, gave, give, told, dad, baby, big, shot, giving, making, wanted, lady, sh*t, f*ck	proponents
2	0.03	reaction, hoping, sick, kids, allergic, eggs, made, egg, chicken, allergy, medicine, tea	allergic reaction
3	0.09	flu, season, people, year, virus, immune, system, shot, epidemic, strain, stay, healthy, spreading, protect, remember	reminders
4	0.04	influenza, pregnant, risk, vaccination, national, recommend, women, immunity, free, safe	risk
5	0.07	free, health, today, clinic, center, giving, insurance, hospital, employees, students, tomorrow, student, public, appointment	unknown
6	0.11	arm, sore, today, hurts, hurt, yesterday, damn,	pain and

		left, feels, side, bad, feel, stupid, throat, pain, ouch, killing, feeling, hurting	distress
7[w]	0.31	flu, sick, time, year, today, feel, good, hope, people, work, shot, cold, week, feeling, glad, day, years, thing	unknown
8	0.07	waiting, cvs, line, walgreens, pharmacy, free, wait, office, give, long, gave, spray, clinic, nasal, giving, people	queues concerns
9	0.19	sick, hate, shots, fuck, shit, flu, needles, damn, today, nervous	fear
10	0.08	Work, office, day, today, free, morning, tomorrow, doctors, doctor, shot, good, school	vaccination places

Table MA3. Theraflu topics (Beta = 0.28)

Topic Number	Alpha	Topic Compositions	Interpretation
1	0.23	soup, chicken, noodle, juice, orange, care, flu, work, sleepy, warm, easier, food, drinkin, spicy, vodka	natural flu remedies
2	0.19	told, chain, hoe, bad, flu, black, sprite, finna, selling, days, fuck, stay, asked, eating, powder, chainz, wack, talk, swear, cold&flu	unknown
3[w]	2.31	flu, cold, sick, tea, drink, drinking, hot, good, night, time, bed, cough, sleep	unknown
4	0.18	kill, symptoms, life, cure, headache, nite, miss, wine, westnile, lord, great, suggest, cloud, tongue, thoughts, aches, trick, human, dying	unknown
5	0.21	girl, whiskey, shot, ill, discontinued, kids, embarrass, happy, fun, fluids, what's, kid, single, beat, money, wit, mixed, listening, awesome, cocktail	unknown

Table MA4. Oseltamivir topics (Beta = 0.96)

Topic Number	Alpha	Topic Compositions	Interpretation
1	1.20	bed, influenza, day, reporting, kinda, make, sleep, works, found, onset, girl, find, call, gave, year, shortage, flu, tested, mentioning, disappointed	unknown
2[w]	5.52	flu, symptoms, feel, day, doctor, prescription, made, meds, fever, taking, sick, prescribe,	prescription of drug

		effects	
3	1.30	report, worse, asap, approved, give, body, coming, prescribed, medical, person, real, flu, cut, thought, surprises, short, dry, goodness, part, official	unknown

Table MA5. Acetaminophen topics (Beta = 0.38)

**Topic Number**	**Alpha**	**Topic Compositions**	**Interpretation**
1	0.73	fever, throat, juice, recommend, drowsy, soup, temp, symptom, strep, spray, orange, tea	natural flu remedies
2[w]	4.08	flu, cold, severe, night, lol, feel, time, cold/flu, feeling, shots, sick, shit, hope	symptoms
3	0.67	pills, test, back, bad, high, stay, gonna, yup, full, cool, knock, guess, tomorrow, multi, crap, hate, pop, wanna, tweeting, duck	unknown

Table MA6. Vitamins topics (Beta = 0.22)

**Topic Number**	**Alpha**	**Topic Compositions**	**Interpretation**
1[w]	0.27	flu, cold, sick, taking, lots, rest, drink, eat, immune, people, stay, feeling, good, healthy	preparedness through vitamins
2	0.17	cold/flu, flu, fever, garlic, throat, tomorrow, stay, warm, hope, health, beat, kind, flu-like, thought, trust, doctor, flu/cold, hoping	unknown
3	0.14	flu, season, it's, plenty, remember, sunrise, tequila, avoid, loading, shot, makes, bad, damn, husband, drinking, refuse, extra, nasty, alrightt	unknown
4	0.08	man, crazy, sniffles, fever, veggies, bad, safely, adding, living, germs, hear, extra, making, starting, foods	unknown
5	0.09	husband, sleep, duh, watch, apple, cayenne, shot, germs, fear, kiwi, mg, due, weather, careful, back, dr, fatty	unknown
